# The Etiology, Pathology, and Treatment of Troublesome Tooth Sockets

**Published:** 1905-04

**Authors:** M. I. Schamberg

**Affiliations:** Philadelphia


### International Dental Journal.

Vol. XXVI. April, 1905. No. 4.

### Original Communications.[1]

### THE ETIOLOGY, PATHOLOGY, AND TREATMENT OF TROUBLESOME TOOTH SOCKETS.[2]

BY M. I. SCHAMBERG, D.D.S., M.D., PHILADELPHIA.

Since man has been made aware of the worth of his teeth and the advisability of retaining them as long as possible, even at the expense of time, money, and a moderate amount of physical pain, the number of unwarranted extractions has been materially reduced. Coincident with this laudable conservation of these important organs, there is noted a tendency toward the retention for too long a period of teeth, the removal of which would prove a far better service to the patient. The wobbling molar with its pyorrhœal socket, the uncured abscessed tooth with its fistulæ, the impacted wisdom-tooth with its readily infected pocket, and the exostosed tooth with its disturbing influence upon the enveloping bone are all examples of cases which frequently come to the extractor at such a late date that a troublesome tooth socket is bound to follow. It is, indeed, unfortunate that just and prompt dis-

[1] The editor and publishers are not responsible for the views of authors of papers published in this department, nor for any claim to novelty, or otherwise, that may be made by them. No papers will be received for this department that have appeared in any other journal published in the country.

[2] Read before the Academy of Stomatology, Philadelphia, November 22, 1904.

crimination cannot always be made between the tooth that will grow well under treatment and the one whose inevitable loss is but a matter of time. It is, however, possible for each and every one of us to make up his mind to cure every abscess, check the pus discharge in every pyorrhœal case, brace every loose tooth, or cause these offending members to be removed lest they menace the health and longevity of their hosts.

Your essayist takes this emphatic stand for the reason that he has observed many cases where the prolonged retention of diseased teeth has not alone endangered the integrity of the surrounding tissues, but has been the cause of constitutional disorders of serious import. The citation of an illustrative case might here be of interest. Mr. H. presented himself for the treatment of an abscess upon the lower second molar tooth. Examination of the mouth revealed a fistula upon the buccal aspect of the alveolus communicating with the distal root. Upon inquiry it was learned that the patient had noticed for two years or more a "small gum-boil" in this region. The usual history was obtained that the abscess filled from time to time and would then burst, discharging its contents into the mouth. During this time the patient had been a sufferer from frequent outbreaks of boils and carbuncles upon the neck and other parts of the body. He was treated both here and abroad for this distressing condition, yet no thought had been given the possible connection between this small alveolar abscess and the constitutional poisoning from which he was suffering. His trip abroad and the cures taken there improved his health, but he still suffered from an occasional boil. An effort made to cure the abscess failed because of an obstruction in the distal canal which interfered with its proper cleansing, whereupon the tooth was removed.

Though a year has elapsed, the patient has not had a recurrence of his boils, and his health is much improved. There is little doubt that this general infection was due to the absorption of pus from the alveolar abscess, and that the patient might have been spared several years of needless suffering, inconvenience, and expense had this tooth been removed a few years earlier. The review of this case could be followed by a number of other cases in which the gastro-intestinal tract and the entire system have suffered from the swallowing of pus formed within the mouth, from faulty mastication rendered so by loose and tender teeth, and from physical exhaustion due to prolonged suffering. Thus far in this paper particular stress has been laid upon the baneful effects of the retention of diseased teeth upon the general health. This is done to emphasize the necessity for removing such teeth unless they respond to treatment within a reasonable period of time. The removal of aching or sore teeth is almost invariably followed by prompt relief. The purpose of this paper is to consider clinically and pathologically the causes of those cases in which relief does not follow the extraction of a tooth. Attention will also be directed to the prevention and treatment of troublesome tooth sockets. For the purpose of systematically dealing with the subject, troublesome tooth sockets might be classified in the following manner:

- Traumatic Sockets.

  - 1. Injury to alveolar process.

  - 2. Laceration of soft tissues.

  - 3. Unremoved ends of roots.

- Dry Sockets.

  - 1. Interference with the formation of blood-clot

    - through drugs.

    - through suppuration.

    - through hæmophilic tendency.

  - 2. Dense alveolar walls

    - exostosis.

    - impaction.

    - peridental inflammation.

  - 3. Infection

    - pre-existing: abscess, pyorrhœa.

    - operative.

    - post-operative.

To adequately consider these various deviations from the normal requires a full understanding of the uneventful extraction of a tooth and healing of the socket without pathological interference. When a tooth is removed no greater local disturbance should be produced than the rupture of the peridental membrane and the pulp at the apex of the tooth, together with a moderate amount of spreading of the process made possible through the normal resiliency of that tissue. The bleeding which takes place should entirely fill the socket of the tooth with a well-formed blood-clot. This clot is supposed to remain and form a matrix for the new cells which spring from the socket wall and rapidly spread until they have replaced the clot and caused its absorption. Most sockets heal in the manner just outlined. It must be remembered, however, that there are many conditions which tend to alter or interfere with this normal process of healing. Teeth do not always possess the ideal conformation, and are therefore not always removed with as little trauma as characterizes the uneventful tooth extraction. Neither are all teeth free from affections which alter the surrounding structures sufficiently to prevent the normal repair. Again, the mouth of many patients and the instruments and hands of many operators are not always free from organisms which are ever ready to invade the socket and work their noxious effects. Lastly, a proportion of patients, however small, come to the extractor at such a time when their lowered state of vitality tends to unfavorably influence the proper healing of the socket.

By *traumatic socket* is meant an injury to the socket produced during the extraction of a tooth in excess of the rupture of the peridental membrane and pulp and slight spreading of the process. The traumatic socket, next to the normal socket, is the one most often met with, but fortunately it seldom proves to be as troublesome as the dry socket.

1. *Injury to the alveolar process* constitutes a frequent variety of traumatic socket. It consists of an excessive spreading, crushing, or fracture of the process. It may be due to the shape of the tooth, as noted in teeth with exostosed, curved, or flaring roots, to an abnormal position of the tooth, to brittleness of the process, to its adherence to the tooth, to the necessity for crushing through the alveolus to dislodge a root, or to carelessness, haste, or incompetence of the operator. This form of traumatism may cause the part to remain tender for some time, though it seldom, unless complicated by other conditions, causes excruciating or unbearable pain. Small forceps, elevators, the surgical bur, care, and skill will often prevent excessive injury to the process. When the alveolus has been fractured, loose pieces should be removed and sharp edges made smooth so as to cause as little irritation as possible.

2. *Laceration of the soft tissues* usually accompanies injury to the process, though it may occur independent of that complication. It consists of tearing and crushing the gum tissue, and may be due to the gum being adherent to the tooth, to the removal of a piece of process with gum attached, to grasping the gum with the forceps, to crushing through it to reach a root, and to the same faults of the operator, as were mentioned in referring to process injury. Here the employment of small forceps, elevators, and the lance will reduce the possibility of laceration to a minimum. The soreness from injury to the gums seldom lasts very long, and the repair takes place with promptitude unless interfered with by continued irritation from projecting process or through suppuration.

3. *Unremoved ends of roots* are always barriers against the normal healing of the socket. Roots become fractured during extraction, through efforts at hasty removal of teeth rather than a slow spreading of the process and a gradual dislodgement of teeth in the direction of least resistance. Exostosed, curved, and frail roots are naturally prone to this mishap. Impaction, brittleness, excessive decay, density of the enveloping bone, and non-yielding process are also conditions which predispose to this injury. Unremoved roots may or may not give trouble, much depending upon whether they contain exposed portions of vital pulp, abscesses at their apices, or other peridental disturbances.

It is always well to be sure of the presence or absence of the ends of roots. This may be accomplished by carefully examining every tooth removed, by the skilful use of the explorer, by swabbing the socket with a styptic so as to check all bleeding, by the use of a good electric mouth-lamp with a mirror to reflect the light into the socket, and by the use of the X-rays when other methods fail. It is always best to remove every portion of every root, lest it immediately or eventually give trouble. *Every root can be removed. The judgment of the operator must decide whether the end justifies the means.* When it is decided to allow a portion of a root to remain until nature makes its removal an easier task the patient should be kept under observation, and upon the first indication of trouble occasioned by the retained root the same should be promptly removed.

By *dry socket* is meant a socket in which the normal blood-clot is absent. This term is a misnomer, for a socket is not necessarily dry though it be devoid of its blood-clot. The name is here used for want of a better means of designating the condition and because of its accepted usage.

1. *Interference with the formation of a blood-clot* is the usual cause of a dry socket, and may follow the use of drugs in the socket, may be brought about through suppuration, or may be the result of bleeders' diathesis. In each instance the clot fails to form and leaves the socket unprotected and open to the reception of saliva and oral débris. Such sockets are usually painful, and can be best relieved by thorough cleansing with peroxide of hydrogen and packing with sterile boric acid or iodoform gauze, or with a tampon of cotton saturated with campho-phenique. This treatment must be applied until the walls of the socket are covered with a layer of healthy granulations, after which time the part is usually able to take care of itself.

In a communication received some time ago from Dr. Louis Jack, in which he requested that I prepare for the INTERNATIONAL DENTAL JOURNAL an article on the subject of troublesome tooth sockets, he says, " Concerning the condition which has been designated ' dry socket,' my observations have led me to consider that it is most frequently caused by the use of such disinfectants immediately after extraction as have stopped the natural hemorrhage and thus prevented the formation of the protective coagulum. The effusion of lymph is also checked.

" This result has repeatedly occurred in healthy and clean mouths, and most frequently after the removal of third molars, more particularly of lower ones. This has been more the case when phenol sodique has been used. The consequence of this unprotected condition of the alveolus renders the process liable to severe irritation, presumably infective. The condition bears some relation to that of compound fracture. If the mouth has been disinfected with dioxide or permanganate of potash, I can see no necessity for the use of active or irritating disinfectants in the sockets. My preference is for the use of the normal aqueous solution of either acetanilide or hydronaphthol in the form of spray. Neither of these interferes with coagulation. The former has the advantage of arresting pain. A solution of two parts of the tincture of calendula to six parts of water, as a spray, is exceedingly useful in mild cases and is especially soothing where laceration takes place."

The substance of the above quotation is deserving of weighty consideration, coming as it does from such a trustworthy and experienced stomatologist as Dr. Jack. His allusion to the destruction of the protective coagulum by the application of phenol preparations and the consequent production of a dry socket is pertinent, and offers food for thought to those who practise the invariable post-operative swabbing of the socket with a disinfectant.

It was upon Dr. Jack's advice that I have long since discontinued this practice, save in such cases in which the existing disease of the socket necessitates active treatment.

2. Dense alveolar walls, such as are formed about exostosed and impacted teeth and those the seat of chronic peridental inflammations, are occasionally found to be the cause of dry sockets. This constitutes probably the most painful variety of troublesome tooth socket, for in such cases there is a total absence of bleeding in the socket and a complete denudation of the socket wall. The cancellated portion of the bone having been rendered dense there is little opportunity for granulations to spring from the socket wall. Such cases can be treated in a similar manner to those in which there is an absence of blood-clot from other causes, but a much longer time is required for the granulations to spread from the edges of the socket. By burring out the dense wall of the socket the cancellated bone is sometimes reached and more rapid healing made possible.

3. Infection of tooth sockets may be due to a pre-existing alveolar abscess or pyorrhœa, to the introduction of germs at the time of the removal of the tooth, and to the migration of organisms into the socket some time after the extraction. Most cases of alveolar pyorrhœa and abscess are immediately benefited by the extraction of teeth involved. There is, however, a small proportion of cases in which the traumatism occasioned by the removal of the affected teeth aggravates the trouble for the time being. In such cases a thorough curettement of the diseased area, followed by cauterization with pure carbolic acid, is usually followed by relief. The socket should be then loosely packed and allowed to heal by gradually filling with granulations. If a clot be allowed to form over an infected area before complete drainage is effected, an extension of the disease is likely to follow. Serious consequences have resulted from infections of the bone brought about in this way. An osteomyelitis so produced calls for an immediate and thorough burring out of the socket with careful antiseptic treatment. Orthoform applied to the socket will relieve the pain when carbolic acid fails.

*Operative infection* may be due to non-sterile instruments and to an unhygienic condition of the mouth. *Post-operative infection* is usually due to the latter cause. The gravity of infection thus produced will depend upon the character and number of the germs, their virulence, and the resistance offered by the tissues. Fortunately, the staphylococcic and streptococcic infections far outnumber the tetanic, the erysipelatous, the syphilitic, and the tubercular, but the bare possibility of such germs gaining access to the system through wounds created by tooth extraction warrants the utmost care as to the sterilization of all instruments, the disinfection of the operator's hands, and the antiseptic treatment of the patient's mouth.

Miller, in the September, 1903, issue of the *Dental Cosmos*, says, "I have found that the secretions of wounds produced by the extraction of teeth examined three or four hours after operation contain vast numbers of leucocytes, and that in many cases they are loaded down with bacteria." Farther on in this laudable contribution to dental literature, entitled "Immunity to Diseases of the Mouth and Teeth," he says, "The protective powers present in the mouth are not to be accounted for by any antiseptic action on the part of the saliva, but rather by the phenomenon of phagocytosis, by the struggle for existence, and probably by certain forces residing in the soft tissues which have not yet been investigated."

These observations made by Miller warrant the conclusion that in the battle which is waged between the white blood-corpuscles and the bacteria-laden saliva the defence against infection could be materially strengthened by the use of mouth-washes which will dispose of a proportion of the invading organisms. Permanganate of potassium 1 in 1000 is the mouth-wash which I usually employ to precede extraction. It is not a pleasant-tasting wash, but is an excellent oxidizer, deodorizer, and disinfectant, and if vigorous rinsing is encouraged the mouth can be rendered fairly sterile in a few moments. Staining resulting from its use can be promptly overcome by hydrogen dioxide, which makes a good supplementary wash because of its combined bleaching and antiseptic properties. I do not recommend permanganate of potassium to patients for use at their homes after extraction because of the deep staining that will follow its prolonged usage. Patients are much more likely to adhere to instructions if advised to use some pleasant mouth-wash, such as borine, listerine, glyco-thymoline, or the wash to which they are accustomed.

All instruments and appliances used during the removal of teeth, such as forceps, elevators, cotton-pliers, mouth-props, etc., should be thoroughly sterilized, preferably by boiling or by immersion in a formalin solution. The operator's hands should be thoroughly cleansed immediately prior to handling the patient, the use of warm water and a non-irritating soap being sufficient except where special precautionary measures are necessary, when the hands should be dipped in a 1 in 1000 bichloride solution or in an equally efficient wash. The extraction of a tooth being a surgical procedure, its performance should be accompanied by similar precautions to those that are employed in general surgery. *In the light of our present knowledge of the prevalence of germs within the oral cavity, the failure to precede and supplement every operation within the mouth which entails the shedding of blood by antiseptic washing or douching should be stamped as unpardonable negligence.*

